# Antioxidant capacity and concentration of redox-active trace mineral in fully weaned intra-uterine growth retardation piglets

**DOI:** 10.1186/s40104-015-0047-7

**Published:** 2015-11-19

**Authors:** Hao Zhang, Yue Li, Tian Wang

**Affiliations:** College of Animal Science and Technology, Nanjing Agricultural University, Weigang 1, Nanjing, 210095 China

**Keywords:** Antioxidant capacity, Intra-uterine growth retardation, Oxidative damage, Piglet, Redox-active trace mineral

## Abstract

**Background:**

The redox status of intra-uterine growth retardation (IUGR) piglets post-weaning has been poorly studied.

**Methods:**

Newborns from twenty-four sows were weighted, weaned at 21 d and fed a starter diet until sampling. Sampling was done at 14 d post-weaning. A piglet was defined as IUGR when its birth weight was 2 SD below the mean birth weight of the total population. At weaning, eighteen piglets with nearly equal body weight from each category (i.e. IUGR or normal birth weight (NBW) piglets) were selected and then allocated to two treatments, consisted of six replicates with each pen having three piglets.

**Results:**

Compared with NBW group, IUGR significantly decreased average daily gain (*P* < 0.001), average daily feed intake (*P* = 0.003), and feed efficiency (*P* < 0.001) of piglets during the first two weeks post-weaning. IUGR decreased the activities of total antioxidant capacity (*P* = 0.019), total superoxide dismutase (T-SOD, *P* = 0.023), and ceruloplasmin (*P* = 0.044) but increased the levels of malondialdehyde (*P* = 0.040) and protein carbonyl (*P* = 0.010) in plasma. Similarly, the decreased activities of T-SOD (*P* = 0.005), copper- and zinc-containing superoxide dismutase (Cu/Zn-SOD, *P* = 0.002), and catalase (*P* = 0.049) was observed in the liver of IUGR piglets than these of NBW piglets. IUGR decreased hepatic Cu/Zn-SOD activity (*P* = 0.023) per unit of Cu/Zn-SOD protein in piglets when compared with NBW piglets. In addition, IUGR piglets exhibited the decreases in accumulation of copper in both plasma (*P* = 0.001) and liver (*P* = 0.014), as well as the concentrations of iron (*P* = 0.002) and zinc (*P* = 0.048) in liver. Compared with NBW, IUGR down-regulated mRNA expression of Cu/Zn-SOD (*P* = 0.021) in the liver of piglets.

**Conclusions:**

The results indicated that IUGR impaired antioxidant capacity and resulted in oxidative damage in fully weaned piglets, which might be associated with the decreased levels of redox-active trace minerals. This study highlights the importance of redox status in IUGR offspring and provides a rationale for alleviating oxidative damage by dietary interventions aiming to supplement trace minerals and to restore redox balance in the future.

## Background

Malnutrition, environmental stress, hypoxia or any other reasons during pregnancy may lead to growth lag and developmental restriction of the fetus, this condition is termed “intra-uterine growth retardation” (IUGR) [1]. Approximately 5-10 % of human fetuses worldwide suffer from IUGR and IUGR fetus has a reduced functional capacity and fewer cells [2]. The latter may be part of a general reduction in cell numbers or a selective trade-off in the development of tissues that are less critical to the body, such as liver, pancreas and intestine [3–5]. Therefore, IUGR exerts a permanent stunting effect on postnatal growth and impairs long-term health in offspring [1].

The pig has been recognized as one of the ideal models for the study of clinic nutrition in humans because of its similar metabolic features, cardiovascular systems, and proportional organ sizes [6]. An increasing number of studies have emerged in recent years to investigate the metabolic changes in IUGR pigs and some novel mechanistic insights have been obtained [5, 7], but few studies have focused on the effect of IUGR on accumulation of trace minerals. Trace minerals play important roles in multiple biological processes and the alteration of local and systemic trace minerals often occurs under chronic inflammation or oxidative stress [8]. Substantial evidence suggests that redox-active trace minerals, such as selenium (Se), iron (Fe), copper (Cu), zinc (Zn) and manganese (Mn), are important modulators of cellular redox status and oxidative stress [8, 9]. However, the levels of these minerals and their alterations in IUGR offspring have not been studied at systems biology levels, especially during the early period post-weaning when the piglet is subjected to several stressors. Therefore, the aim of the present study was to investigate the effects of IUGR on redox status and levels of redox-active trace minerals in fully weaned piglets in order to provide a rationale for establishing new feeding strategies of IUGR offspring.

## Materials and methods

### Experimental design, diets and management

Experimental protocols were permitted by the Institutional Animal Care and Use Committee of Nanjing Agricultural University (NJAU-CAST-2014-179).

Twenty-four pregnant sows (Landrace × Yorkshire) with similar parity (second or third) were given a commercial diet during pregnancy. At birth, birth weight and sex of each newborn piglet (Duroc × (Landrace × Yorkshire)) were recorded. A piglet was defined as IUGR when its birth weight was 2 SD below the mean birth weight of the total population [10]. In each litter, one male IUGR piglet with birth weight of 0.95 ± 0.04 kg and one normal same-sex littermates with birth weight of 1.58 ± 0.04 kg were chosen. At weaning (21 d of age), eighteen piglets with nearly equal body weight from each category (i.e. IUGR or normal birth weight (NBW) piglets) were selected and subsequently transferred to the weaner unit for experiment. Piglets were then allocated to two treatments, consisted of six pen replicates with each pen having three piglets. Both IUGR and NBW piglets were fed a commercial diet for 14 d. The composition and nutrition level of the diet was given in Table 1, which was formulated according to the National Research Council (2012) to meet the nutrient requirements of the piglets [11]. Piglets had free access to food and water until the day of sampling.

**Table 1:** Composition and nutrient level of the diet (as-fed basis)

Items	Contents
Ingredients, %	
Maize	40
Broken rice	15
Fermented soybean meal	10
Dehulled soybean meal	6
Spray-dried animal plasma	5
Whey powder	7
Fish meal	4
Sugar	4.5
Glucose	3
Soybean oil	1.5
L-lysine-HCl, 98 %	0.3
L-methionine	0.15
L-threonine	0.2
L-tryptophan	0.05
L-leucine	0.1
L-isoleucine	0.05
L-valine	0.05
Salt	0.3
Limestone	1.0
CaHPO_4_	0.8
Vitamin mixture^a^	0.2
Mineral mixture^b^	0.8
Total	100
Nutrient level, %	
Crude protein	20.2
Digestible energy, Mcal/kg	3.40
Total calcium	0.85
Total phosphorus	0.70
Available phosphorus	0.44
Digestible lysine	1.45
Digestible methionine + cystine	0.79
Digestible threonine	0.81
Digestible tryptophan	0.23
Digestible isoleucine	0.74
Digestible leucine	1.55
Digestible valine	0.89
Analyzed nutrient levels	
Calcium, %	0.85
Total phosphorus, %	0.69
Fe, mg/kg	199.8
Zn, mg/kg	122.5
Cu, mg/kg	127.1
Mn, mg/kg	63.2

^a^The vitamin mixture supplied the following per kg complete diet: Vitamin A, 9,000 IU; Vitamin D_3_, 3,000 IU; Vitamin E, 30 IU; Vitamin K_3_, 3 mg; Vitamin B_1_, 3 mg; Vitamin B_2_, 8 mg; Vitamin B_6_, 5 mg; Vitamin B_12_, 0.04 mg; biotin, 0.3 mg; pantothenic acid, 20 mg; niacin, 45 mg; folic acid, 2 mg; choline chloride, 450 mg

^b^The mineral mixture supplied the following per kg complete diet: Fe (from ferrous sulfate), 110 mg; Cu (from copper sulfate), 120 mg; Zn (from zinc sulfate), 100 mg; Mn (from manganese sulfate), 50 mg; I (from calcium iodate), 0.9 mg; Se (from manganese sulfate), 0.3 mg

### Sample collection

At 35 d of age, six piglets with nearly equal body weight from each treatment (one piglet per pen) were selected. Heparinized blood samples were drawn by jugular vein puncture, and then centrifuged at 2,000 *g* for 10 min at 4 °C. The acquired plasma was stored at −80 °C for further determination. All piglets were killed by electrical stunning and exsanguination, the liver was then rapidly removed. Liver samples were collected from the left lateral lobe within 5 min, which were rapidly frozen in liquid nitrogen and stored at −80 °C for further analyses.

### Growth performance measurement

Pig body weight and feed intake were recorded and measured on a pen basis at weaning and sampling to calculate average daily gain (ADG), average daily feed intake (ADFI), and feed efficiency (FE).

### Antioxidant capacity measurement

The activities of total superoxide dismutase (T-SOD), copper- and zinc-containing superoxide dismutase (Cu/Zn-SOD), glutathione peroxidase (GPX), total antioxidant capacity (T-AOC) and ceruloplasmin (CP), and the concentrations of malondialdehyde (MDA) and protein carbonyl in the plasma, the activities of T-AOC, T-SOD, Cu/Zn-SOD, GPX and catalase (CAT), and concentrations of MDA, protein carbonyl and total protein in the liver were determined using colorimetric methods with spectrophotometer, respectively. The assay kits used in the assay was purchased from the Nanjing Jiancheng Institute of Bioengineering (Nanjing, Jiangsu, China) and the assay was conducted following the instructions of the kits. The results in the liver were normalised against total protein concentration in each sample for inter-sample comparison.

Briefly, the activities of T-SOD and Cu/Zn-SOD were assayed at 550 nm by use of the nitrite method as described by Ōyanagui [12], Cu/Zn-SOD activity was determined on the chloroform/ethanol (3:5, v/v) extract. One unit of T-SOD or Cu/Zn-SOD activity was defined as the amount of SOD required to produce 50 % inhibition of the rate of nitrite production at 37 °C in 1 min. Hafeman et al.’s dithio-nitro benzene method was used to determine GPX activity [13]. The GPX activity was assayed at 412 nm using glutathione as a substrate by measuring the glutathione decrease in the enzymatic reaction (except for the effect of the non-enzymatic reaction). One unit of GPX activity was defined as the amount of enzyme depleting 1 μmol of glutathione at 37 °C in 1 min. The activity of T-AOC was measured at 520 nm by the method of ferric reducing-antioxidant power assay [14]. One unit of T-AOC was defined as the amount that increased the absorbance by 0.01 at 37 °C in 1 min. Ceruloplasmin activity was determined according to the method of Martinez-Subiela et al. [15]. One unit of ceruloplasmin activity was defined as the amount of ceruloplasmin consuming 1 μmol of substrate (*o*-dianisidine dihydrochloride) at 37 °C in 1 min. The method of CAT assay is based upon alteration of hydrogen peroxide optical density, depending on enzymatic decomposition of hydrogen peroxide [16]. One unit of CAT activity was defined as the amount of CAT consuming 1 μmol hydrogen peroxide at 405 nm for 1 s. The concentration of MDA was measured according to the thiobarbituric acid reactive substance (TBARS) method [17]. Content of protein carbonyl was determined by derivatisation using dinitrophenylhydrazine as reported previously [18].

### Cu/Zn-SOD protein analysis

The level of Cu/Zn-SOD protein in the liver were determined using an enzyme linked immunosorbent assay (ELISA) kit (pig-specific, No. 026002, Shanghai Enzyme linked Biotechnology Co., Ltd., Shanghai, China) according to the manufacturer's instruction. The samples were assayed in triplicate. The ELISA results were obtained by a microplate reader at the light length of 450 nm. Hepatic Cu/Zn-SOD protein was expressed as picogram per milligram (pg/mg) of total protein. In addition, the activity of Cu/Zn-SOD was normalised against Cu/Zn-SOD protein level in each sample for inter-sample comparison.

### Trace mineral determination

The levels of Se, Zn, Fe, Mn, and Cu were analysed by inductively coupled plasma mass spectrometry (ICP–MS) using an Optimal 2100DV instrument (Perkin-Elmer-Sciex, Norwalk, NY, USA) equipped with a standard spray chamber (Ryoton) and a cross-flow nebuliser. Briefly, 2 ml of plasma or 1 g of a liver sample were firstly dissolved in an appropriate amount of 65 % nitric acid and perchloric acid (3:1, v/v) and then digested on a heating block, after which they were diluted with ultra-pure water to a final volume of 25 mL. Blanks and standard solutions were prepared. After that, samples were analyzed immediately.

### Total RNA isolation and mRNA quantification

Messenger RNA abundance was determined according to method described by Zhang et al. [5]. RNA was isolated using Trizol Reagent (TaKaRa Biotechnology, Dalian, Liaoning, China) from snap-frozen liver sample using the manufacturer’s protocol. RNA integrity was checked on 1 % agarose gel with ethidium bromide staining. The RNA concentration and purity were determined from OD260/280 readings (ratio > 1.8) using a NanoDrop ND-1000 UV spectrophotometer (NanoDrop Technologies, Wilmington, DE, USA). After determining the RNA concentration, 1 μg of total RNA was reverse-transcribed into cDNA using PrimeScript™ RT Reagent Kit (TaKaRa Biotechnology, Dalian, Liaoning, China) according to the manufacturer’s guidelines. Real-time PCR was performed on an ABI StepOnePlus™ Real-Time PCR System (Applied Biosystems, Grand Island, NY, USA) according to the manufacturer’s instructions. The primers were designed using the Primer-Blast (http://www.ncbi.nlm.nih.gov). The primer sequences for the target and reference genes (copper- and zinc-containing superoxide dismutase (*Cu/Zn-SOD*), manganese-containing superoxide dismutase (*Mn-SOD*), glutathione peroxidase 1 (*GPX1*), thioredoxin (*TXN*), thioredoxin 2 (*TXN2*) and β-actin) are given in Table 2. Briefly, the reaction mixture was prepared using 2 μL of cDNA (50 μg/mL), 0.4 μL of forward primer (20 μmol/L), 0.4 μL of reverse primer (20 μmol/L), 10 μL of SYBR Premix Ex Taq™ (TaKaRa Biotechnology, Dalian, Liaoning, China), 0.4 μL of ROX Reference Dye (TaKaRa Biotechnology, Dalian, Liaoning, China) and 6.8 μL of double-distilled water. Each sample was tested in duplicate. RT-qPCR consisted of a pre-run at 95 °C for 30 s and 40 cycles of denaturation at 95 °C for 5 s, followed by a 60 °C annealing step for 30 s. The conditions of the melting curve analysis were as follows: one cycle of denaturation at 95 °C for 10 s, followed by an increase in temperature from 65 °C to 95 °C at a rate of 0.5 °C/s. The relative levels of mRNA expression were calculated using the 2^-ΔΔC^_T_ method [19], in which the β-actin gene was amplified as an internal standard.

**Table 2:** Sequences for real-time PCR primers

Genes^a^	GenBank ID	Primer sequence (5’→3’), sense/antisense	Length, bp	Amplification efficiency
*Cu/Zn-SOD*	NM_001190422.1	CATTCCATCATTGGCCGCAC/TTACACCACAGGCCAAACGA	118	1.01
*Mn-SOD*	NM_214127.2	CAAGAAGGGGCACCACGTT/CTCAGGGGACGCAAGAACTG	70	1.00
*GPX1*	NM_214201.1	CCTCAAGTACGTCCGACCAG/GTGAGCATTTGCGCCATTCA	85	0.98
*TXN*	NM_214313.2	CTGCCAAGATGGTGAAGCAG/CGTGGCTGAGAAATCGACCA	98	1.00
*TXN2*	NM_001243705.1	GACGACAGAAGTGCCCTTGA/CTGAGCCATCTCCCAGCAAC	78	1.03
*β-actin*	DQ178122	TCTGGCACCACACCTTCT/TGATCTGGGTCATCTTCTCAC	114	0.99

^a^ *Cu/Zn-SOD* copper- and zinc-containing superoxide dismutase, *Mn-SOD* manganese-containing superoxide dismutase, *GPX1* glutathione peroxidase 1, *TXN* thioredoxin, *TXN2* thioredoxin 2

### Statistical analysis

Data were analyzed by independent-samples T tests using the SPSS statistical software (Ver.16.0 for windows, SPSS Inc., Chicago, IL, USA). The differences were considered to be significant at *P* < 0.05. *P* values between 0.05 and 0.10 were considered as a trend. All data are presented as group means and standard error.

## Results

### Growth performance

Compared with NBW group (Table 3), IUGR piglets exhibited significantly decreased body weight at both weaning and sampling (*P* < 0.001). The similar effect was also observed for ADG (*P* < 0.001), ADFI (*P* = 0.003) and FE (*P* < 0.001) in IUGR piglets during the first two weeks post-weaning.

**Table 3:** Effect of intra-uterine growth retardation on growth performance of weaned piglets

Items^a^	IUGR	NBW	*P* values
BW, kg			
At weaning	5.31 ± 0.08	7.05 ± 0.08	<0.001
At sampling	7.34 ± 0.23	10.70 ± 0.26	<0.001
ADG, g/d	144.73 ± 12.82	260.51 ± 13.44	<0.001
ADFI, g/d	256.94 ± 25.80	382.62 ± 20.00	0.003
FE^b^, g/g	0.57 ± 0.01	0.68 ± 0.01	<0.001

^a^ *IUGR* intra-uterine growth retardation group, *NBW* normal birth weight group, *ADG* average daily gain, *ADFI* average daily feed intake, *FE* feed efficiency

^b^FE was calculated by dividing the ADG by its ADFI

### Antioxidant capacity

IUGR piglets had significantly lower plasma T-AOC (*P* = 0.019), T-SOD (*P* = 0.023) and CP (*P* = 0.044) activities, whereas increased MDA (*P* = 0.040) and protein carbonyl (*P* = 0.010) contents than those of NBW piglets (Table 4). IUGR significantly decreased the activities of T-SOD (*P* = 0.005), Cu/Zn-SOD (*P* = 0.002), and CAT (*P* = 0.049) in the liver of piglets compared with NBW piglets. A tendency towards a decreased protein expression of Cu/Zn-SOD (*P* = 0.053) was observed in the liver of IUGR piglets. IUGR decreased the Cu/Zn-SOD activity (*P* = 0.023) per unit of Cu/Zn-SOD protein in the liver of piglets when compared with NBW piglets. IUGR tended to decrease hepatic T-AOC activity (*P* = 0.085) in piglets. There was no significant difference in GPX activity among the groups (*P* > 0.10).

**Table 4:** Effect of intra-uterine growth retardation on redox status of weaned piglets

Items^a^	IUGR	NBW	*P* values
Plasma			
T-AOC, U/mL	3.80 ± 0.37	5.06 ± 0.23	0.019
T-SOD, U/mL	97.92 ± 3.09	117.39 ± 6.04	0.023
GPX, U/mL	246.97 ± 9.48	243.86 ± 7.97	0.807
CP, U/L	124.72 ± 5.47	148.68 ± 8.86	0.044
MDA, nmol/mL	3.03 ± 0.42	1.89 ± 0.15	0.040
Protein carbonyl, nmol/mL	0.32 ± 0.02	0.25 ± 0.01	0.010
Liver			
T-AOC, U/mg protein	4.38 ± 0.26	5.45 ± 0.50	0.085
T-SOD, U/mg protein	65.99 ± 3.63	86.62 ± 4.37	0.005
Cu/Zn-SOD, U/mg protein	45.67 ± 2.76	63.01 ± 3.29	0.002
GPX, U/mg protein	124.05 ± 6.64	142.49 ± 10.28	0.163
CAT, U/mg protein	57.08 ± 5.47	71.27 ± 6.58	0.049
MDA, nmol/mg protein	0.91 ± 0.11	0.72 ± 0.09	0.200
Protein carbonyl, nmol/mg protein	5.02 ± 1.46	3.29 ± 0.61	0.300
Cu/Zn-SOD, pg/mg protein	28.08 ± 1.34	31.81 ± 1.05	0.053
Cu/Zn-SOD, U/pg Cu/Zn-SOD protein	14.00 ± 0.90	17.48 ± 0.94	0.023

^a^ *IUGR* intra-uterine growth retardation group, *NBW* normal birth weight group, *T-AOC* total antioxidant capacity, *T-SOD* total superoxide dismutase, *Cu/Zn-SOD* copper- and zinc-containing superoxide dismutase, *GPX* glutathione peroxidise, *CP* ceruloplasmin, *MDA* malondialdehyde, *CAT* catalase

### Redox-active trace mineral concentration

As indicated in Table 5, compared with NBW piglets, IUGR resulted in an obviously decreased Cu (*P* = 0.001) concentration in the plasma of piglets. Similarly, a tendency towards a decreased level of plasma Fe (*P* = 0.079) was also observed in IUGR piglets. However, IUGR piglets showed a tendency for increased Se concentration in plasma (*P* = 0.084). In addition, IUGR significantly decreased the concentrations of Zn (*P* = 0.048), Fe (*P* = 0.002) and Cu (*P* = 0.014) in the liver of piglets. No obvious difference was found for the concentration of Mn between the groups (*P* > 0.10).

**Table 5:** Effect of intra-uterine growth retardation on the concentrations of redox-active trace minerals of weaned piglets

Items^a^	IUGR	NBW	*P* values
Plasma, mg/L			
Se	0.37 ± 0.01	0.31 ± 0.03	0.084
Zn	0.33 ± 0.02	0.36 ± 0.03	0.342
Fe	6.92 ± 0.63	8.51 ± 0.51	0.079
Mn	0.29 ± 0.01	0.32 ± 0.01	0.102
Cu	1.68 ± 0.07	2.08 ± 0.05	0.001
Liver, mg/kg wet weight			
Se	0.46 ± 0.06	0.66 ± 0.13	0.196
Zn	58.64 ± 4.27	73.17 ± 4.85	0.048
Fe	102.96 ± 7.51	158.23 ± 11.13	0.002
Mn	3.04 ± 0.16	3.24 ± 0.20	0.453
Cu	10.75 ± 0.65	14.41 ± 1.06	0.014

^a^ *IUGR* intra-uterine growth retardation group, *NBW* normal birth weight group, *Se* selenium, *Zn* zinc, *Fe* iron, *Mn* manganese, *Cu* copper

### Gene expression

The expression levels of genes related to hepatic redox status are summarised in Table 6. IUGR piglets had a lower mRNA expression of *Cu/Zn-SOD* compared with NBW piglets. However, no alterations were observed in the mRNA abundances of *Mn-SOD*, *GPX1*, *TXN* and *TXN2* among the groups (*P* > 0.10).

**Table 6:** Effect of intra-uterine growth retardation on hepatic gene expression related to redox status of weaned piglets

Items^a^	IUGR	NBW	*P* values
*Cu/Zn-SOD*	0.84 ± 0.03	1.23 ± 0.14	0.021
*Mn-SOD*	1.02 ± 0.11	1.02 ± 0.05	0.973
*GPX1*	0.94 ± 0.18	1.18 ± 0.12	0.290
*TXN*	0.95 ± 0.06	1.07 ± 0.07	0.233
*TXN2*	0.96 ± 0.07	1.11 ± 0.15	0.417

^a^ *IUGR* intra-uterine growth retardation group, *NBW* normal birth weight group, *Cu/Zn-SOD* copper- and zinc-containing superoxide dismutase, *Mn-SOD* manganese-containing superoxide dismutase, *GPX1* glutathione peroxidase 1, *TXN* thioredoxin, *TXN2* thioredoxin 2

## Discussion

Several studies have demonstrated that IUGR piglets exhibited poor growth performance compared with their heavier counterparts during the post-weaning period [3, 5, 20, 21], and these findings are basically in agreement with the results of the present study in which IUGR piglets had decreased ADG, ADFI, and FE during the first two weeks post-weaning. The factors, such as inadequate nutrient intake, digestive shortcomings, compromised intermediate metabolism or hormonal imbalances, contribute to the poor growth performance of IUGR offspring [3–5, 20, 21]. However, few studies paid attention on the roles of antioxidant capacity and redox-active trace minerals exerted in this process.

Oxidative stress occurs when there is an imbalance between production of free radicals and reactive metabolites, so-called reactive oxygen species (ROS), and their elimination by protective mechanisms, referred to as antioxidants [22]. Oxidative damage is believed to play a major role in the pathogenesis of several serious conditions in IUGR infants and to increase the risk of metabolic syndrome in adulthood [23–25]. At the cellular level, the concentrations of ROS (such as superoxide anions, hydrogen peroxide and hydroxyl radicals) exceeding the antioxidant protection levels can cause widespread damage to DNA, proteins and endogenous lipids [26]. To date, descriptions of changes of the redox status during the pigs’ lifetime are rather fragmentary [27, 28]. In the present study, increased MDA and protein carbonyl accumulation were observed in fully weaned IUGR piglets, which are similar to the results of Liu et al. [29] who found higher concentrations of MDA and protein carbonyl in the liver of IUGR piglets, which might be associated with the decreased Mn-SOD activity. Superoxide dismutase can reduce the radical superoxide to form hydrogen peroxide and oxygen [30]. The SOD family includes three different enzymes: cytosolic Cu/Zn-SOD, mitochondrial Mn-SOD and extracellular SOD [31]. Subsequently, GPX or CAT removes hydrogen peroxide by oxidizing reduced glutathione to oxidized glutathione [32]. In addition, vitamins, biological antioxidants, CP and uric acid also play important roles in scavenging the oxygen free radicals. Here, IUGR-induced oxidative injury was found to reduce the T-AOC activity in both plasma and liver, which is in accordance with the findings of Michiels et al. [21]. The T-AOC reflects the levels of non-enzymatic antioxidant defense system and antioxidant enzyme. In contrast to the results of Michiels et al. [21], we failed to observe a decrease in GPX activity in the plasma of IUGR piglets. However, a significantly depressed T-SOD and Cu/Zn-SOD activities were found in IUGR piglets. These observations pointed to a lower antioxidant capacity of fully weaned IUGR pigs upon ROS and oxidants when oxidative stress occurred. Moreover, the mRNA abundance of *Cu/Zn-SOD* was down-regulated by IUGR in the liver of piglets, which may provide an explanation for the decreased protein expression of hepatic Cu/Zn-SOD.

Using the state-of-the-art ICP–MS technique, a recent study found strong associations between ionomic profiles and metabolic abnormalities [33]. Likewise, the redox status is closely related to the concentrations of redox-active trace minerals in fully weaned IUGR piglets. In the present study, we found that IUGR piglets had lower concentrations of Fe in both plasma and liver, coupled with a decrease in hepatic CAT activity. Although the role of Fe in heme for oxygen transport is well known, its role for redox status as a component of peroxidase and CAT should not be overlooked. Schultze and Kuiken reported that Fe deficiency could lead to a decreased hepatic CAT activity [34]. Macdougall also found that the activities of GPX and CAT increased in iron-deficient children following Fe treatment [35].

Copper serves many roles in the body and often it acts as an enzyme activator. Notably, Cu is known to be important in changing the valence of Fe for binding to transferrin and ferritin [36]. The lower Fe accumulation in the IUGR piglets may also partially result from the simultaneously decreased Cu concentration. Ceruloplasmin as an acute-phase protein binds 90 to 95 % of the Cu in the blood and is also responsible for the transport of Cu [37]. Hill et al. reported that neonatal pigs with Cu deficiency showed no serum CP activity [38]. Therefore, there was a positive relation between the decreased Cu content and the lower CP activity in IUGR piglets. Ceruloplasmin is not just involved in the transport of Cu, but it is an antioxidant enzyme. The decreased plasma CP activity also confirmed the poor antioxidant capacity of fully weaned IUGR piglets.

Additionally, previous study found a lower concentration of both Zn and Cu in IUGR placentas [39]. Zinc has a vital role in a wide range of biological functions through its participation as a constituent of more than 100 metalloenzyme systems. The free-radical scavenging mechanisms may be impaired in the case of Zn deficiency. The activity of SOD is not only activated by the expression of SOD-related genes but also mediated by other factors, such as Zn and Cu levels [40]. Zinc as a component of Cu/Zn-SOD is closely inter-related to antioxidant functions [40]. Besides, decrease of both Zn and Cu concentrations may be caused by the increase of Se content in the plasma of IUGR piglets, as Se appears to mimic the behavior of Zn and is opposite to that of Cu [41]. Their higher Se concentration might play a positive role in maintaining Se-dependent GPX activity at the level comparable with GPX activity in NBW piglets.

## Conclusions

The present study indicates that the metabolism of redox-active trace minerals such as Fe, Cu, and Zn in the IUGR piglets may be inferior, which most likely due to simultaneously reduced feed intake. The results may offer a possible explanation for the impaired antioxidant system in the IUGR piglets. This study may help to guide nutrition interventions for IUGR offspring to alleviate oxidative damage during the early period after weaning.

## Abbreviations

CAT: catalase
CP: ceruloplasmin
Cu: copper
*Cu/Zn-SOD*: copper- and zinc-containing superoxide dismutase
Fe: iron
GPX: glutathione peroxidise
*GPX1*: glutathione peroxidase 1
ICP–MS: inductively coupled plasma mass spectrometry
IUGR: intra-uterine growth retardation group
MDA: malondialdehyde
Mn: manganese
*Mn-SOD*: manganese-containing superoxide dismutase
NBW: normal body weight group
ROS: reactive oxygen species
Se: selenium
SOD: superoxide dismutase
T-AOC: total antioxidant capacity
*TXN*: thioredoxin
*TXN2*: thioredoxin 2
Zn: zinc
